# Potential influence of carbonic anhydrase 9 genetic variants and expression levels on the progression of diabetic retinopathy

**DOI:** 10.7150/ijms.131188

**Published:** 2026-03-30

**Authors:** I-Chia Liang, Hsiang-Wen Chien, Kai Wang, Chia-Yi Lee, Ying-Erh Chou, Shun-Fa Yang

**Affiliations:** 1 Department of Ophthalmology, Tri-Service General Hospital, Taipei, Taiwan.; 2 Department of Ophthalmology, College of Medicine, National Defense Medical Center, Taipei, Taiwan.; 3 Department of Ophthalmology, Cathay General Hospital, Taipei, Taiwan.; 4 Departments of Ophthalmology, Sijhih Cathay General Hospital, New Taipei City, Taiwan.; 5 School of Medicine, College of Medicine, Fu Jen Catholic University, New Taipei, Taiwan.; 6 School of Medicine, National Tsing Hua University, Hsinchu, Taiwan.; 7 Institute of Medicine, Chung Shan Medical University, Taichung, Taiwan.; 8 School of Medicine, Chung Shan Medical University, Taichung, Taiwan.; 9 Department of Medical Research, Chung Shan Medical University Hospital, Taichung, Taiwan.

**Keywords:** ARPE-19 cells, carbonic anhydrase IX, diabetic retinopathy, hypoxia, single nucleotide polymorphism, vascular dysfunction

## Abstract

Diabetic retinopathy (DR) represents one of the most common microvascular complications of diabetes, and proliferative diabetic retinopathy (PDR) is the most vision-threatening form. Carbonic anhydrase IX (CA9), a hypoxia-inducible enzyme, has been implicated in several pathological processes, but its involvement in DR has not been clarified. In this study, three *CA9* single nucleotide polymorphisms (rs3829078, rs2071676, and rs1048638) were genotyped in diabetic patients with and without DR. Clinical characteristics were compared between groups, and expression analyses were conducted using public databases and ARPE-19 cells under hyperglycemic and hypoxic conditions. No significant association was observed between *CA9* variants and DR susceptibility in the overall cohort. However, among patients aged ≤60 years, carriers of the rs1048638 C/A and C/A + A/A genotypes exhibited a significantly increased risk of PDR. Expression quantitative trait locus (eQTL) data from the GTEx database revealed higher CA9 mRNA expression in tissues harboring the rs1048638 A allele. Moreover, both transcriptomic data and *in vitro* experiments demonstrated upregulation of CA9 in ARPE-19 cells exposed to high glucose and hypoxia. These findings suggest that the rs1048638 polymorphism may modulate CA9 expression and contribute to PDR development in younger diabetic patients through hypoxia- and glucose-related mechanisms. Collectively, our findings suggest that CA9 may serve as a potential risk biomarker associated with the progression of DR, particularly in younger patients.

## 1. Introduction

Diabetic retinopathy (DR) is one of the most common microvascular complications of diabetes mellitus. Non-proliferative diabetic retinopathy (NPDR) represents the early stage of the disease, whereas proliferative diabetic retinopathy (PDR) is an advanced form characterized by pathological neovascularization and is a leading cause of vision loss in adults 1. The pathogenesis of DR is complex and multifactorial, involving a dynamic interplay among metabolic, hemodynamic, and genetic factors 2, 3. The transition from NPDR to PDR is driven by progressive retinal ischemia, which induces the release of proangiogenic mediators, particularly vascular endothelial growth factor (VEGF), thereby promoting the formation of fragile, leaky neovessels 4. Identifying patients at high risk for progression from NPDR to PDR is crucial, as those who develop PDR face a markedly increased likelihood of severe, vision-threatening complications and incur substantially higher medical costs 5. Although clinical factors such as diabetes duration, poor glycemic control, and coexisting vascular complications (e.g., nephropathy or chronic non-healing ulcers) have been recognized as risk factors for DR progression, they do not fully account for the onset or severity of the disease 6, 7. Genetic variability likely contributes to interindividual differences in DR susceptibility and progression, even among patients with similar clinical and metabolic profiles.

In PDR, hypoxia-inducible factor-1 alpha (HIF-1α), a transcription factor activated under hypoxic conditions, serves as a central regulator of genes mediating cellular adaptation to low oxygen tension 8, 9. In response to hypoxia, HIF-1α directly binds to the promoter region of the VEGF gene and also upregulates the expression of carbonic anhydrase IX (CA9), a gene implicated in several cancer types 10-13. Although hypoxia is the primary inducer of CA9 expression, proinflammatory cytokines such as interleukin-1 and -6 (IL-1, IL-6), tumor necrosis factor-alpha (TNF-α), and transforming growth factor-beta (TGF-β) have also been shown to modulate CA9 expression 14. These cytokines likewise play crucial roles in the pathophysiology of PDR 15.

Carbonic anhydrases (CAs) constitute a large family of zinc metalloenzymes that catalyze the reversible hydration of carbon dioxide, generating bicarbonate and protons. The CA9 gene encodes carbonic anhydrase IX, a transmembrane protein that contributes to pH regulation and cell adhesion, processes critical for angiogenesis and the modulation of the tumor microenvironment 16. Genetic variations in angiogenesis- and vascular-related genes have been shown to influence individual susceptibility to DR 17. Single nucleotide polymorphisms (SNPs) within the CA9 gene have been associated with the development and progression of various cancers, potentially by altering gene expression, protein conformation, or molecular interactions 18-21. While previous genetic studies have highlighted the roles of oxidative stress, apoptosis, inflammation, and autophagy in DR pathogenesis, the precise molecular mechanisms underlying disease initiation and progression remain unclear 22. Given the established associations between CA9 polymorphisms and diverse clinicopathological features, it is plausible that these genetic variants may also modulate susceptibility to DR and influence its progression—an area that warrants further investigation.

## 2. Materials and Methods

### 2.1. Subjects

A total of 1,534 diabetic patients from the department of endocrinology at Chung Shan Medical University Hospital (Taichung, Taiwan) were recruited for this study. Prior to initiation, ethical approval was obtained from the Institutional Review Board of Chung Shan Medical University Hospital (IRB No. CS2-22190), and written informed consent was secured from all participants. Clinical data were retrieved from medical records, including the duration of diabetes, presence or absence of DR, glycated hemoglobin (HbA1c) levels, renal function, and lipid profiles. DR was defined as the presence of diabetes-related retinal microvascular abnormalities and was classified as NPDR or PDR. PDR was defined by the presence of retinal or optic disc neovascularization as previously described 23. For the non-DR control group, the absence of DR was confirmed through dilated fundus examinations conducted by experienced ophthalmologists.

### 2.2. Specimen collection and genomic DNA extraction

Peripheral whole blood samples were collected in ethylenediaminetetraacetic acid (EDTA)-containing tubes, centrifuged immediately, and stored at -80°C 24. Genomic DNA was extracted using the QIAamp DNA Blood Mini Kit (Qiagen, Valencia, CA, USA) according to the manufacturer's protocol. Extracted DNA was dissolved in TE buffer (10 mM Tris, 1 mM EDTA; pH 7.8), and its concentration was determined by spectrophotometric measurement at 260 nm. The DNA samples were stored at -20 °C and used as templates for subsequent polymerase chain reaction (PCR) analyses 25.

### 2.3. Selection and Analysis of CA9 Genetic Polymorphisms

Among the more than 30 known single nucleotide polymorphisms (SNPs) in the CA9 gene, three SNPs, rs2071676 (+201, G/A) in exon 1, rs3829078 (+1081, A/G) in exon 7, and rs1048638 (+1584, C/A) in the 3′ untranslated region (UTR) of exon 11, were selected based on their potential functional relevance and previous reports indicating their involvement in hypoxia-related pathways and tumor vascular invasion 20, 26, 27. Genotyping of these polymorphisms was performed using the ABI StepOne™ Real-Time PCR System (Applied Biosystems, Foster City, CA, USA) and TaqMan SNP Genotyping Assays. Data were analyzed with SDS software version 3.0 (Applied Biosystems) 28.

### 2.4. Bioinformatics Analysis

To evaluate the association between the rs1048638 SNP and CA9 gene expression, data from the Genotype-Tissue Expression (GTEx) database were analyzed for correlations in aorta, coronary artery, and esophageal mucosal tissues 29. Additionally, gene expression profiles were analyzed using datasets (GSE233164 and GSE151610) obtained from the Gene Expression Omnibus (GEO) database, as these datasets include gene expression data from samples relevant to diabetic retinopathy, enabling comparative analysis of gene expression patterns.

### 2.5. Cell culture

The ARPE-19 human retinal pigment epithelial cell line was obtained from the Bioresources Collection and Research Center (BCRC), Food Industry Research and Development Institute (Hsinchu, Taiwan). Cells were cultured in Dulbecco's Modified Eagle's Medium/Nutrient Mixture F-12 (DMEM/F12) supplemented with 10% fetal bovine serum (FBS) and 1% penicillin-streptomycin. Cells were maintained at 37 °C in a humidified atmosphere containing 5% CO₂. For experimental treatments, cells were exposed to high-glucose conditions (25 mM glucose) for 24 hours. Hypoxia was induced by incubation in a hypoxic chamber with 1% O₂ for 24 hours, depending on the experimental protocol.

### 2.6. Western blotting

ARPE-19 cells were exposed to either hypoxic or high-glucose conditions for 24 hours, washed with PBS, and lysed using ice-cold lysis buffer as previously described 30. Total protein concentrations were determined, and 20 μg of protein from each sample was separated by SDS-PAGE and transferred onto a PVDF membrane. The membrane was blocked with 5% nonfat milk and incubated with anti-CA9 primary antibody at 4 °C overnight, followed by incubation with horseradish peroxidase (HRP)-conjugated secondary antibodies (Dako Corporation, Carpinteria, CA, USA) 31. Protein bands were visualized using an enhanced chemiluminescence detection system, and band intensities were quantified using ImageJ software.

### 2.7. Real time PCR

Total RNA was extracted using the Total RNA Mini Kit (Geneaid, New Taipei City, Taiwan), and complementary DNA (cDNA) was synthesized using the High-Capacity cDNA Reverse Transcription Kit (Applied Biosystems, Foster City, CA, USA). Real-time PCR amplification was conducted as described in previous studies, following standard protocols 32.

### 2.8. Statistical analysis

A *p*-value of < 0.05 was considered statistically significant. Differences in demographic and clinical characteristics between the NPDR and PDR groups were assessed using the Mann-Whitney U test and Fisher's exact test. Adjusted odds ratios (AORs) and 95% confidence intervals (CIs) for the associations between genotypes and DR risk were calculated using multiple logistic regression, controlling for potential confounders. Because subgroup analyses were performed according to age, the statistical power of these stratified analyses may be relatively limited due to reduced sample sizes and should therefore be interpreted with caution. All statistical analyses were performed using SAS software (version 9.1; SAS Institute, Cary, NC, USA) for Windows.

## 3. Results

### 3.1. Characteristics of the study participants

The clinical and laboratory characteristics of the two diabetic groups, patients without diabetic retinopathy (non-DR) and those with diabetic retinopathy (DR), are summarized in Table 1. No statistically significant differences were observed between the groups in terms of age, age at diabetes onset, or sex distribution. However, the duration of diabetes was significantly longer in the DR group compared to the non-DR group (9.05 vs. 11.30 years, *p* < 0.001). With respect to laboratory parameters, patients in the DR group exhibited significantly higher levels of glycated hemoglobin (HbA1c) and serum creatinine, as well as a lower estimated glomerular filtration rate (eGFR), compared to those in the non-DR group. No significant differences were found in lipid profile parameters, including total cholesterol, high-density lipoprotein (HDL), low-density lipoprotein (LDL), and triglycerides (TG).

### 3.2. Association of CA9 gene polymorphisms with diabetic retinopathy

The genotype distributions of three CA9 SNPs (rs3829078, rs2071676, and rs1048638) between the non-DR and DR groups are presented in Table 2. No significant differences were observed in the frequencies of these SNPs between the two groups. To further investigate the relationship between these polymorphisms and disease severity, the DR group was stratified into NPDR and PDR subgroups. As shown in Table 3, no statistically significant differences in the genotype distributions of the three CA9 SNPs were identified among the non-DR, NPDR, and PDR groups.

Subsequently, the cohort was stratified by age into two groups: patients aged >60 years and those aged ≤60 years. As shown in Table 4, no significant associations were found between the *CA9* polymorphisms and DR status in either age subgroup. However, when both age and disease severity were considered simultaneously, a significant association emerged. Among patients aged ≤60 years, carriers of the rs1048638 C/A genotype, as well as those with the combined C/A + A/A genotypes, showed a significantly higher risk of developing PDR compared with individuals carrying the C/C genotype. The adjusted odds ratio (AOR) for the C/A genotype was 1.509 (95% CI: 1.191-1.913, *p* = 0.035), and the 95% confidence interval does not include 1.0, supporting the statistical significance of this association. Similarly, individuals with the combined C/A + A/A genotypes had an increased risk of PDR, with an AOR of 1.475 (95% CI: 1.018-2.138, *p* = 0.040) (Table 5).

### 3.3. Functional and clinical relevance of CA9 rs1048638 in diabetic retinopathy

**Figure 1:**
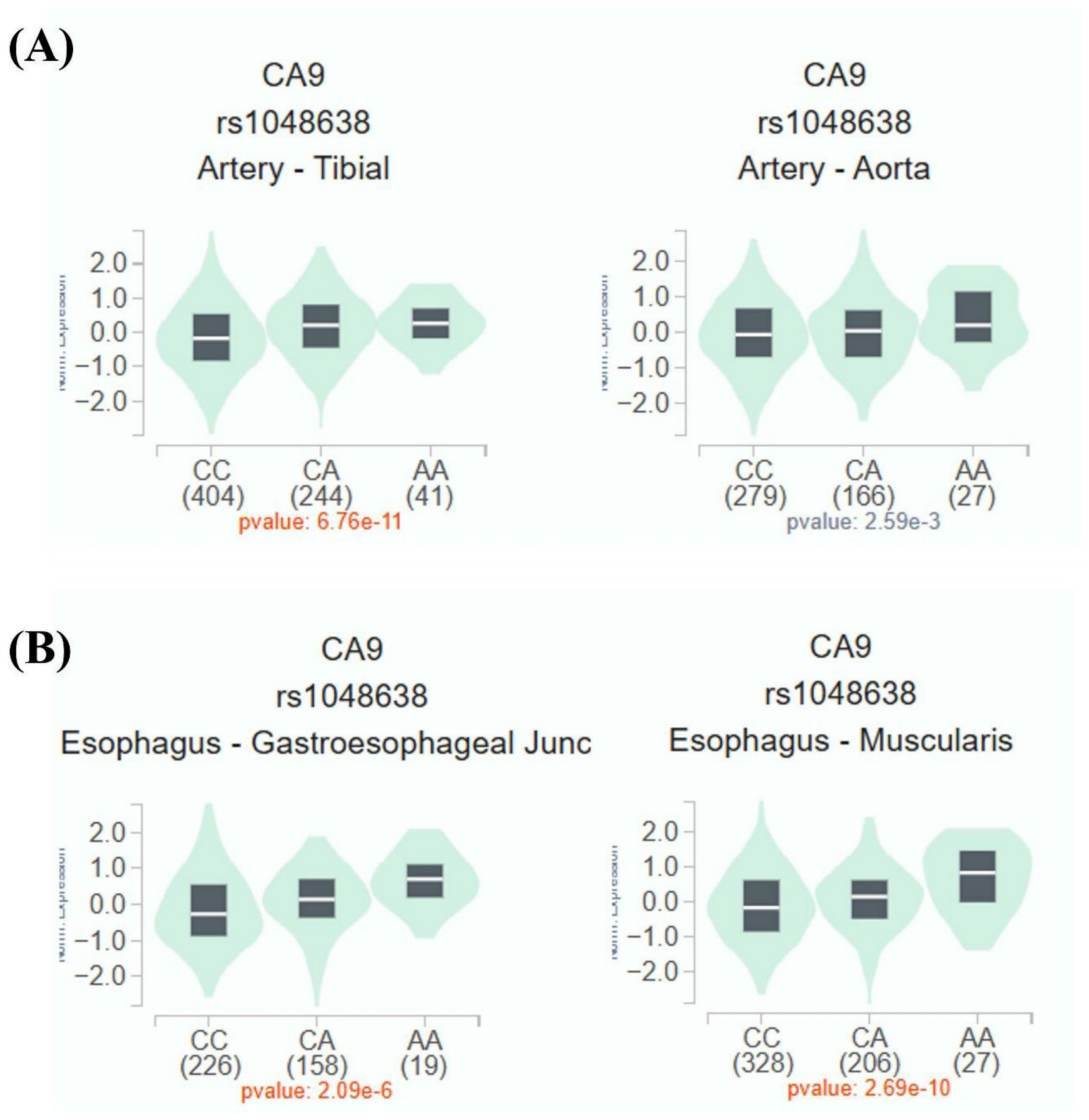
** rs1048638 regulates CA9 expression.** Expression quantitative trait locus (eQTL) analysis of rs1048638 in aortic and esophageal tissues was performed using data from the GTEx portal.

**Figure 2:**
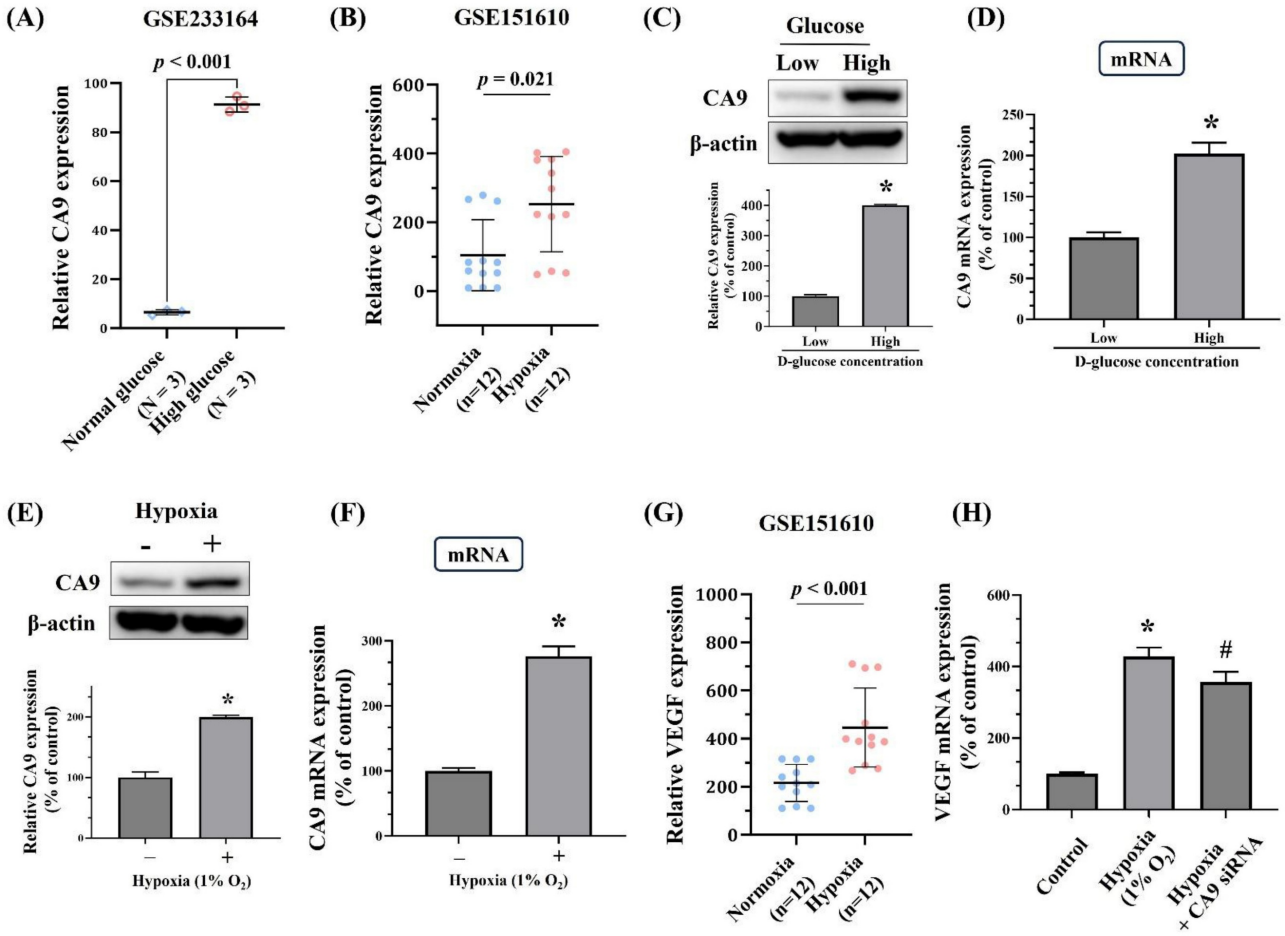
** Validation of CA9 and VEGF gene expression in ARPE-19 cells using datasets from the Gene Expression Omnibus (GEO) and *in vitro* assay.** (A) CA9 expression was significantly upregulated in ARPE-19 cells treated with high glucose (dataset: GSE233164). (B) Comparison of CA9 expression in ARPE-19 cells under normoxic and hypoxic conditions (dataset: GSE151610). (C-D) Effects of high-glucose treatment on CA9 protein and mRNA levels in ARPE-19 cells were analyzed by Western blotting and real-time PCR. (E-F) Effects of hypoxic stress (1% O₂) on CA9 protein and mRNA levels in ARPE-19 cells were assessed by Western blotting and real-time PCR. (G) VEGF expression under hypoxic stress in ARPE-19 cells (dataset: GSE151610). (H) ARPE-19 cells were transfected with CA9-specific siRNA and exposed to hypoxia (1% O₂). CA9 knockdown led to a significant reduction in VEGF mRNA levels compared with hypoxia-exposed cells. Data are presented as mean ± SD. *p < 0.001 versus control. # *p* < 0.001 versus hypoxia condition.

Given the observed association between rs1048638 and PDR, additional analyses using publicly available datasets were performed to explore the potential functional significance of this PDR-associated SNP. As shown in Figure 1A and 1B, expression quantitative trait locus (eQTL) analysis from the Genotype-Tissue Expression (GTEx) database demonstrated differential CA9 mRNA expression in aortic and esophageal tissues among individuals with distinct rs1048638 genotypes. To further elucidate the functional role of CA9 in ARPE-19 cells, we examined CA9 expression under high-glucose and hypoxic conditions using data obtained from the Gene Expression Omnibus (GEO) database. As shown in Figure 2A, data from GSE233164 revealed a significant upregulation of CA9 expression in ARPE-19 cells exposed to high glucose. Similarly, analysis of the GSE151610 dataset indicated that CA9 expression was markedly increased under hypoxic stress conditions (Figure 2B). The findings from the GEO datasets were further validated by our *in vitro* experiments. As shown in Figures 2C-2F, both CA9 protein and mRNA levels were significantly elevated in ARPE-19 cells following high-glucose treatment (Figure 2C-D) and hypoxic exposure (Figure 2E-F). To explore the downstream functional consequences of CA9 upregulation, we evaluated VEGF expression under hypoxic conditions and performed CA9 knockdown experiments. Analysis of the GSE151610 dataset indicated that VEGF expression was markedly increased under hypoxic stress (Figure 2G). Moreover, CA9-specific siRNA was used to silence CA9 in ARPE-19 cells under hypoxia, resulting in a significant reduction of VEGF mRNA levels (Figure 2H). These results suggest that CA9 contributes to the regulation of angiogenic signaling in retinal cells during hypoxic stress.

## 4. Discussion

The development of DR is influenced by a complex interplay of genetic predisposition and acquired lifestyle and metabolic factors 33, 34. In the present study, we observed that younger diabetic patients (aged ≤60 years) carrying the rs1048638 C/A or C/A + A/A genotypes of the *CA9* gene had a significantly increased risk of developing PDR. Data from the GTEx database indicated elevated CA9 mRNA expression in tissues harboring the A allele of rs1048638, suggesting a regulatory role of this polymorphism in gene expression. Furthermore, transcriptomic analyses from the GEO database and *in vitro* experiments in ARPE-19 cells demonstrated upregulation of CA9 expression under high-glucose and hypoxic conditions. These findings collectively support a potential role for CA9 polymorphism in the pathogenesis of DR, particularly PDR, in younger diabetic individuals, potentially mediated through glucose- and hypoxia-responsive pathways.

The initiation and progression of DR are believed to be driven by retinal capillary dysfunction and subsequent tissue hypoxia, which stimulate angiogenesis and neovascularization 35, 36. Carbonic anhydrases (CAs), including CA9, have been increasingly implicated in the pathophysiology of diabetic complications, and inhibitors of CA activity have shown therapeutic potential 37, 38. The L-arginine/nitric oxide (NO) signaling axis, which is often disrupted in diabetes, plays a key role in maintaining vascular homeostasis and is tightly linked to hypoxic stress in the retina 39, 40. Reduced NO bioavailability contributes to impaired vasodilation, decreased retinal blood flow, and aggravated hypoxia. Moreover, an imbalance between NO and reactive oxygen species (ROS) exacerbates oxidative stress, damaging retinal neurons and microvasculature. Dysregulation of this pathway also influences inflammatory signaling, further contributing to retinal injury 39, 41, 42.

Recent studies have identified the activation of the arginase pathway via nicotinamide adenine dinucleotide phosphate (NADPH) oxidase (NOX) as a major contributor to retinal vascular dysfunction in DR. NOX activation is known to modulate CA9 expression and, conversely, CA9 may influence NOX activity 39. Notably, CA9 has been implicated in the regulation of the L-arginine/NO pathway and has been found to be altered in patients with type 1 diabetes 43. Higher CA9 protein levels have been associated with more rapid DR progression in individuals with type 1 diabetes, compared to those with slower disease trajectories 40. Experimental studies further demonstrate that CA inhibitors improve oxygenation in the optic nerve and retina 44, 45, and topical CA inhibitors have been shown to enhance blood flow velocity in major retinal arteries 36. However, our experiments focused on ARPE-19 cells, DR pathogenesis also involves vascular endothelial cells, which play a central role in microvascular damage and neovascularization. Future studies should investigate the functional consequences of CA9 variants in retinal endothelial cells and other relevant cell types to clarify their contribution to DR progression.

Age is an important modifier in the development of diabetic complications. It reflects a multifactorial process shaped by genetic, metabolic, and environmental factors. Younger individuals with diabetes may have increased susceptibility to hypoxic injury due to higher metabolic demand, early-stage diabetic nephropathy, or impaired systemic oxygen delivery 46. In this study, the age cutoff of 60 years, which has been commonly used in epidemiological studies of diabetic complications to distinguish younger and older patient populations, was applied to explore potential age-dependent genetic effects. In this context, genetic variants such as CA9 polymorphisms, previously implicated in tumorigenesis and hypoxia adaptation, may influence physiological responses to hypoxia and modulate disease risk across age groups 47, 48. In our study, the rs1048638 C/A and A/A genotypes were associated with an increased risk of PDR among patients aged ≤60 years. However, given the moderate effect size (AOR ≈ 1.5) and the exploratory nature of the stratified analyses, these findings should be interpreted with caution. Multiple testing considerations were taken into account when evaluating the statistical significance of the stratified results, and further validation in larger and independent cohorts will be necessary to confirm the clinical relevance of this association.

In our study, the rs1048638 C/A and A/A genotypes were associated with a significantly increased risk of PDR in patients aged ≤60 years. This polymorphism may influence CA9 mRNA stability or translational efficiency, thereby affecting protein expression and cellular responses to hypoxic stress. In cervical cancer, the A allele of rs1048638 has been reported to disrupt the binding site for microRNA-34a (miR-34a), leading to elevated CA9 expression and potentially promoting tumor progression 49, 50. Similar associations have been observed in urothelial carcinoma, where the CA/AA genotype correlates with more aggressive tumor behavior 49, as well as in colorectal and hepatocellular carcinomas 19, 27. These effects are likely mediated through impaired miR-34a-mediated regulation. MiR-34a plays a multifaceted role in diabetes, where it regulates vascular integrity, pancreatic β-cell function, glucose metabolism, and other key physiological processes 51. In retinal endothelial cells, miR-34a suppresses mitochondrial function and biogenesis, enhancing susceptibility to oxidative stress 52. Under hyperglycemic conditions, miR-34a dysregulation contributes to vascular dysfunction and promotes stress-induced premature senescence, thereby accelerating retinal microvascular damage and cellular aging 53. However, the present study did not investigate the interaction between rs1048638 and miR-34a in ARPE-19 cells or other retinal cell types. Therefore, the precise molecular mechanism by which this polymorphism may influence CA9 expression in the context of diabetic retinopathy remains unclear. Future studies are warranted to explore whether the rs1048638 variant affects miR-34a binding and CA9 regulation in retinal cells under hyperglycemic or hypoxic conditions.

Taken together, our findings suggest a potential modulatory effect of CA9 polymorphisms on the progression of DR in younger patients. However, several limitations should be acknowledged. First, the study was conducted in a single ethnic population, which may limit the generalizability of the findings. Second, gene expression analyses relied in part on the GTEx database, which comprises non-diabetic, non-retinal tissues; thus, the inferred expression patterns may not fully reflect the diabetic retina. Finally, the present study did not investigate the molecular mechanisms by which CA9 variants, such as rs1048638, may influence gene expression via microRNA interactions, including miR-34a. Future studies with larger, multiethnic cohorts, longitudinal designs, and mechanistic analyses in retinal cells are warranted to validate these findings and elucidate the functional role of CA9 polymorphisms in DR pathogenesis.

In conclusion, this study provides evidence that the CA9 rs1048638 polymorphism is associated with an increased risk of proliferative diabetic retinopathy in younger patients with diabetes. Individuals aged ≤60 years carrying the C/A or A/A genotypes demonstrated a significantly higher likelihood of developing PDR. Public transcriptomic datasets and *in vitro* experiments confirmed that CA9 expression is upregulated under hyperglycemic and hypoxic conditions, supporting its role in DR pathophysiology. The involvement of CA9 in hypoxia adaptation, the L-arginine/NO signaling pathway, and miR-34a regulation further underscores its potential contribution to retinal vascular dysfunction. These findings suggest a possible gene-environment interaction that warrants further exploration through well-powered, longitudinal studies across diverse populations. Ultimately, CA9 may serve as a candidate biomarker or therapeutic target for DR, particularly in younger at-risk individuals.

## Figures and Tables

**Table 1:** Clinical and laboratory characteristics of diabetic patients with and without retinopathy.

Variable	Non-Diabetic Retinopathy (N=875)	Diabetic Retinopathy (N=659)	p value
Age (years)	60.88 ± 12.29	61.99 ± 12.07	0.078
Age of onset (years)	51.83 ± 11.55	50.69 ± 11.50	0.057
Male gender [n (%)]	473 (54.1%)	350 (53.1%)	0.713
Duration of diabetes (years)	9.05 ± 7.29	11.30 ± 8.13	<0.001
HbA1c [% (mmol/mol)]	7.07 ± 1.30	7.45 ± 1.42	<0.001
Serum creatinine [mg/dL]	0.97 ± 0.74	1.29 ± 1.38	<0.001
Glomerular filtration rate [ml/min]	82.04 ± 32.88	74.11 ± 34.29	<0.001
Total cholesterol [mmol/L]	160.37 ± 45.32	162.40 ± 43.45	0.381
HDL cholesterol [μmol/L]	45.58 ± 12.15	45.56 ± 14.21	0.979
LDL cholesterol [μmol/L]	86.10 ± 30.52	85.20 ± 31.68	0.577
Triglycerides, [μmol/L]	144.30 ± 206.52	144.07 ± 121.86	0.980

**Table 2:** Adjusted odds ratios (AORs) and 95% confidence intervals (CIs) for the association between CA9 genotypic frequencies and diabetic retinopathy.

Variable	No Diabetic Retinopathy (N=875)	Diabetic Retinopathy (N=659)	AOR (95% CI)	p value
**rs3829078**				
AA	807 (92.2%)	614 (93.2%)	1.000	
AG	68 (7.8%)	44 (6.7%)	0.743 (0.491-1.123)	0.158
GG	0 (0%)	1 (0.1%)	---	---
AG+GG	68 (7.8%)	45 (6.8%)	0.875 (0.712-1.074)	0.200
**rs2071676**				
GG	216 (24.7%)	160 (24.3%)	1.000	
GA	464 (53.0%)	339 (51.4%)	0.963 (0.745-1.244)	0.773
AA	195 (22.3%)	160 (24.3%)	1.126 (0.833-1.522)	0.441
GA+AA	659 (75.3%)	499 (75.7%)	1.005 (0.890-1.135)	0.930
**rs1048638**				
CC	764 (87.3%)	577 (87.6%)	1.000	
CA	107 (12.2%)	78 (11.8%)	0.978 (0.709-1.350)	0.895
AA	4 (0.5%)	4 (0.6%)	1.324 (0.303-5.792)	0.709
CA+AA	111 (12.7%)	82 (12.4%)	0.995 (0.850-1.166)	0.952

The adjusted odds ratio (AOR) with their 95% confidence intervals were estimated by multiple logistic regression models after controlling for the duration of diabetes, HbA1c, serum creatinine levels and glomerular filtration rate.

**Table 3:** Adjusted odds ratios (AORs) and 95% confidence intervals (CIs) for the association between CA9 genotypic frequencies and non-proliferative or proliferative diabetic retinopathy.

	No Diabetic Retinopathy (N=875)	Non-Proliferative Diabetic Retinopathy (N=515)	AOR (95% CI)	Proliferative Diabetic Retinopathy (N=144)	AOR (95% CI)
**rs3829078**					
AA	807 (92.2%)	482 (93.6%)	1.000	132 (91.7%)	1.000
AG	68 (7.8%)	32 (6.2%)	0.728 (0.466-1.138)	12 (8.3%)	0.938 (0.464-1.898)
GG	0 (0%)	1 (0.2%)	---	0 (0.0%)	---
AG+GG	68 (7.8%)	33 (6.4%)	0.869 (0.697-1.083)	12 (8.3%)	0.938 (0.464-1.898)
**rs2071676**					
GG	216 (24.7%)	126 (24.5%)	1.000	34 (23.6%)	1.000
GA	464 (53.0%)	266 (51.7%)	0.984 (0.750-1.291)	73 (50.7%)	0.901 (0.556-1.457)
AA	195 (22.3%)	123 (23.8%)	1.109 (0.805-1.529)	37 (25.7%)	1.226 (0.708-2.120)
GA+AA	659 (75.3%)	389 (75.5%)	1.010 (0.888-1.149)	110 (76.4%)	0.997 (0.795-1.251)
**rs1048638**					
CC	764 (87.3%)	456 (88.5%)	1.000	121 (84.0%)	1.000
CA	107 (12.2%)	55 (10.7%)	0.885 (0.624-1.257)	23 (16.0%)	1.450 (0.847-2.484)
AA	4 (0.5%)	4 (0.8%)	1.597 (0.371-6.866)	0 (0.0%)	---
CA+AA	111 (12.7%)	59 (11.5%)	0.954 (0.804-1.132)	23 (16.0%)	1.187 (0.908-1.552)

The adjusted odds ratio (AOR) with their 95% confidence intervals were estimated by multiple logistic regression models after controlling for the duration of diabetes, HbA1c, serum creatinine levels and glomerular filtration rate.

**Table 4:** Adjusted odds ratios (AORs) and 95% confidence intervals (CIs) for the association between CA9 genotypic frequencies and diabetic retinopathy across different age groups.

	Age ≤ 60 years			Age > 60 years		
	No Diabetic Retinopathy (N=384)	Diabetic Retinopathy (N=271)	AOR (95% CI)	No Diabetic Retinopathy (N=491)	Diabetic Retinopathy (N=388)	AOR (95% CI)
**rs3829078**						
AA	353 (91.9%)	254 (93.7%)	1.000	454 (92.5%)	360 (92.8%)	1.000
AG	31 (8.1%)	17 (6.3%)	0.659 (0.342-1.269)	37 (7.5%)	27 (7.0%)	0.817 (0.478-1.395)
GG	0 (0%)	0 (0.0%)	---	0 (0%)	1 (0.2%)	---
AG+GG	31 (8.1%)	17 (6.3%)	0.659 (0.342-1.269)	37 (7.5%)	28 (7.2%)	0.925 (0.710-1.206)
**rs2071676**						
GG	94 (24.5%)	68 (25.1%)	1.000	122 (24.8%)	92 (23.7%)	1.000
GA	209 (54.4%)	137 (50.6%)	0.882 (0.594-1.309)	255 (51.9%)	202 (52.1%)	1.036 (0.738-1.452)
AA	81 (21.1%)	66 (24.3%)	1.172 (0.734-1.870)	114 (23.3%)	94 (24.2%)	1.117 (0.751-1.661)
GA+AA	290 (75.5%)	203 (74.9%)	0.981 (0.814-1.183)	369 (75.2%)	296 (76.3%)	1.030 (0.877-1.209)
**rs1048638**						
CC	341 (88.8%)	230 (84.9%)	1.000	423 (86.2%)	347 (89.4%)	1.000
CA	42 (10.9%)	40 (14.8%)	1.292 (0.792-2.109)	65 (13.2%)	38 (9.8%)	0.787 (0.509-1.216)
AA	1 (0.3%)	1 (0.3%)	2.168 (0.132-35.572)	3 (0.6%)	3 (0.8%)	1.014 (0.179-5.730)
CA+AA	43 (11.2%)	41 (15.1%)	1.144 (0.898-1.458)	68 (13.8%)	41 (10.6%)	0.893 (0.722-1.104)

The adjusted odds ratio (AOR) with their 95% confidence intervals were estimated by multiple logistic regression models after controlling for the duration of diabetes, HbA1c, serum creatinine levels and glomerular filtration rate.

**Table 5:** Adjusted odds ratios (AORs) and 95% confidence intervals (CIs) for the association between CA9 genotypic frequencies and non-proliferative or proliferative diabetic retinopathy in patients under 60 years of age.

	No Diabetic Retinopathy (N=384)	Non-Proliferative Diabetic Retinopathy (N=216)	AOR (95% CI)	Proliferative Diabetic Retinopathy (N=55)	AOR (95% CI)
**rs3829078**					
AA	353 (91.9%)	203 (94.0%)	1.000	51 (925.7%)	1.000
AG	31 (8.1%)	13 (6.0%)	0.669 (0.334-1.343)	4 (7.3%)	0.623 (0.181-2.142)
GG	0 (0%)	0 (0.0%)	---	0 (0.0%)	---
AG+GG	31 (8.1%)	13 (6.0%)	0.669 (0.334-1.343)	4 (7.3%)	0.623 (0.181-2.142)
**rs2071676**					
GG	94 (24.5%)	57 (26.4%)	1.000	11 (20.0%)	1.000
GA	209 (54.4%)	107 (49.5%)	0.844 (0.558-1.277)	30 (54.5%)	1.112 (0.488-2.532)
AA	81 (21.1%)	52 (24.1%)	1.119 (0.685-1.827)	14 (25.5%)	1.527 (0.598-3.901)
GA+AA	290 (75.5%)	159 (73.6%)	0.959 (0.789-1.166)	44 (80.0%)	1.106 (0.747-1.638)
**rs1048638**					
CC	341 (88.8%)	187 (86.6%)	1.000	43 (78.2%)	1.000
CA	42 (10.9%)	28 (13.0%)	1.216 (0.720-2.054)	12 (21.8%)	**1.509 (1.191-1.913)^a^**
AA	1 (0.3%)	1 (0.4%)	2.579 (0.157-42.445)	0 (0.0%)	---
CA+AA	43 (11.2%)	29 (13.4%)	1.115 (0.861-1.444)	12 (21.8%)	**1.475 (1.018-2.138)^b^**

The adjusted odds ratio (AOR) with their 95% confidence intervals were estimated by multiple logistic regression models after controlling for the duration of diabetes, HbA1c, serum creatinine levels and glomerular filtration rate. ^a^p=0.035 ^b^p=0.040
